# The effects and safety of omega-3 fatty for acute lung injury: a systematic review and meta-analysis

**DOI:** 10.1186/s12957-020-01916-6

**Published:** 2020-09-03

**Authors:** Zhongjie Huang, Jianming Zheng, Wencheng Huang, Meihao Yan, Liyue Hong, Yuancheng Hong, Runnv Jin, Xincheng Huang, Hongtao Fan, Huiling Chen, Heping Yang, Weiping Su, Xiaoping Huang

**Affiliations:** 1 Department of Respiratory Medicine, Quanzhou Strait Hospital, No.180 Huayuan Road, Fengze District, Quanzhou, 362000 Fujian Province China; 2 Department of Infection, Quanzhou Strait Hospital, No.180 Huayuan Road, Fengze District, Quanzhou, 362000 Fujian Province China

**Keywords:** Omega-3 fatty, Acute lung injury, Treatment, Meta-analysis, Review

## Abstract

**Background:**

Several randomized controlled trials (RCTs) have compared the treatment of acute lung injury (ALI) with omega-3 fatty, yet the results remained inconsistent. Therefore, we attempted this meta-analysis to analyze the role of omega-3 fatty in the treatment of ALI patients.

**Methods:**

We searched PubMed databases from inception date to October 31, 2019, for RCTs that compared the treatment of ALI with or without omega-3 fatty. Two authors independently screened the studies and extracted data from the published articles. Summary mean differences (MD) with 95% confidence intervals (CI) were calculated for each outcome by fixed- or random-effects model.

**Results:**

Six RCTs with a total of 277 patients were identified, of whom 142 patients with omega-3 fatty acid treatment and 135 patients without omega-3 fatty treatment. Omega-3 fatty treatments significantly improve the PaO_2_ (MD = 13.82, 95% CI 8.55–19.09), PaO_2_/FiO_2_ (MD = 33.47, 95% CI 24.22–42.72), total protein (MD = 2.02, 95% CI 0.43–3.62) in ALI patients, and omega-3 fatty acid treatments reduced the duration of mechanical ventilation (MD = − 1.72, 95% CI − 2.84 to − 0.60) and intensive care unit stay (MD = − 1.29, 95% CI − 2.14 to − 0.43) in ALI patients.

**Conclusions:**

Omega-3 fatty can effectively improve the respiratory function and promote the recovery of ALI patients. Future studies focused on the long-term efficacy and safety of omega-3 fatty use for ALI are needed.

## Background

Acute lung injury (ALI) is a very common kind of critically ill disease in the clinic, which is caused by various intrapulmonary and external factors such as severe trauma, shock, acidosis, or serious infection [1, 2]. If left untreated, it may progress into acute respiratory distress syndrome (ARDS) with high mortality [3]. In the past decade, great progress has been made in the management of patients with ALI and ARDS. However, the mortality of ALI/ARDS is still high with a range of 40 to 31% [4, 5]. In addition to infection control, low tidal volume protective lung mechanical ventilation and nutritional treatment of ARDS/ALI play an important role in the treatment strategies [6–8], especially the provision of calories by fat nutrition is a key part [9].

At present, the fat emulsions used in clinical practice are mostly based on soybean oil. Soybean oil-based fat emulsions are rich in long-chain fat emulsions, which can affect granulocyte activity under severe stress such as trauma and infection, resulting in impaired immune function and increased lipid peroxidation, which in turn increases organ function damage [10, 11]. This tendency to increase the inflammatory response often makes the use of soybean oil-based fat emulsions in a dilemma clinically. In recent years, omega-3 fatty acids have attracted much attention due to their ability to reduce inflammation and regulate immunity. It has been reported [12, 13] that it can inhibit inflammation and regulate immunity in many diseases. It has good effects in the treatment of severe patients such as sepsis and systemic inflammatory response syndrome [14, 15]. Compared to traditional fat emulsions, omega-3 fatty shows its superiority in nutritional therapy for critically ill patients [16]. However, the studies on the effects of omega-3 fatty for the treatment of ALI are still lacking.

Based on literature review, we have found that there are several randomized controlled trials (RCTs) focusing on the omega-3 fatty in the treatment of ALI, yet the results have remained inconsistent. Furthermore, there are very few related meta-analyses on the role of omega-3 fatty acids in the treatment of ALI; further investigations are needed. Therefore, we attempted to conduct this meta-analysis to evaluate the efficacy and safety of omega-3 fatty in the treatment of ALI, to provide insights into the clinical management of ALI.

## Methods

We attempted to conduct and report this meta-analysis in compliance with the Preferred Reporting Items for Systematic Reviews and Meta-Analyses (PRISMA) [17].

### Literature search

Relevant RCTs on the role of omega-3 fatty in the treatment of ALI were screened and identified. We searched PubMed, EMBASE, Cochrane library, China National Knowledge Infrastructure (CNKI), Wanfang Database, and China Biomedical Literature Database (CBM) for relevant trials published in English or Chinese up to October 31, 2019. The following search terms were used: “omega-3; fish oil; ω-3; acute lung injury; ALI; acute respiratory distress syndrome; ARDS.” We adopted those search terms with combined Boolean operators “AND” or “OR” in every database. Besides, relevant systematic reviews and meta-analyses from those databases were identified, and their bibliographies were scrutinized for further relevant trials, as were those of the RCTs included in the review.

### Eligibility criteria

Two investigators independently reviewed the identified articles. The inclusion criteria for RCTs were as follows: (1) RCT design, (2) comparative analysis of ALI treatment with or without omega-3 fatty, and (3) the details of omega-3 fatty treatment and related outcomes were reported. The exclusion criteria were as follows: (1) case reports, reviews, editorial comments, meeting abstracts, and articles without applicable data; (2) studies with insufficient data, such as missing the standard deviation (SD); and (3) considerable overlaps between the authors and research centers among the published literature.

### Study selection

The search results were imported into the software of Endnote X7 for literature management. Two authors independently reviewed the title, abstract, or descriptors of the identified studies and excluded the studies that clearly did not meet the inclusion criteria. After excluding duplicate and apparently irrelevant studies, the full text of the remaining studies was reviewed to assess eligibility for inclusion. Any disagreements were resolved by discussion or by asking an independent third opinion.

### Data collection

Two authors independently extracted the data from each study with a standardized data extraction checklist, which included the study characteristics (e.g., first author’s name, publication year, journal, country where the study was conducted), characteristics of included study subjects (e.g., number of participants, age, gender distribution), details of omega-3 fatty treatment intervention, outcome variables (e.g., follow-up period), and study conclusion. Outcomes were extracted preferentially by intention to treat (ITT) at the end of follow-up. Quantitative data were extracted to calculate effect sizes. Data on effect size that could not be obtained directly were recalculated whenever possible or contacted the original authors for data. Any discrepancy was resolved by further consensus.

### Risk of bias assessment

Two authors independently assessed the methodological quality of the included studies for major potential sources of bias using the Cochrane Collaboration’s risk of bias tool, which includes the method of random sequence generation, allocation concealment, blinding of participants and personnel, blinding of outcome assessment, incomplete outcome data, selective reporting, and other sources of bias. We evaluated the methodological quality of every included RCT by rating each criterion as low, high, or unclear risk of bias. Any disagreements were resolved through further discussion whenever necessary.

### Statistical analysis

The Review Manager Version 5.3 software was used for data analysis. All the data syntheses and interpretations were also performed by two authors independently to ensure the accuracy of the results. Binary outcomes were presented as Mantel–Haenszel-style odds ratios (OR) with 95% confidence intervals (CIs). Continuous outcomes were reported as mean differences (MDs). A fixed-effect model was adopted in cases of homogeneity (*P* value of *χ*^2^ test > 0.10 and *I*^2^ < 50%), whereas a random-effect model was used in cases of obvious heterogeneity (*P* value of *χ*^2^ test > 0.10 and *I*^2^ ≥ 50%). Publication bias was evaluated by using funnel plots, and asymmetry was assessed by conducting Egger regression test. The statistical significance level was 0.05 in this present study.

## Results

### Study selection

**Fig. 1:**
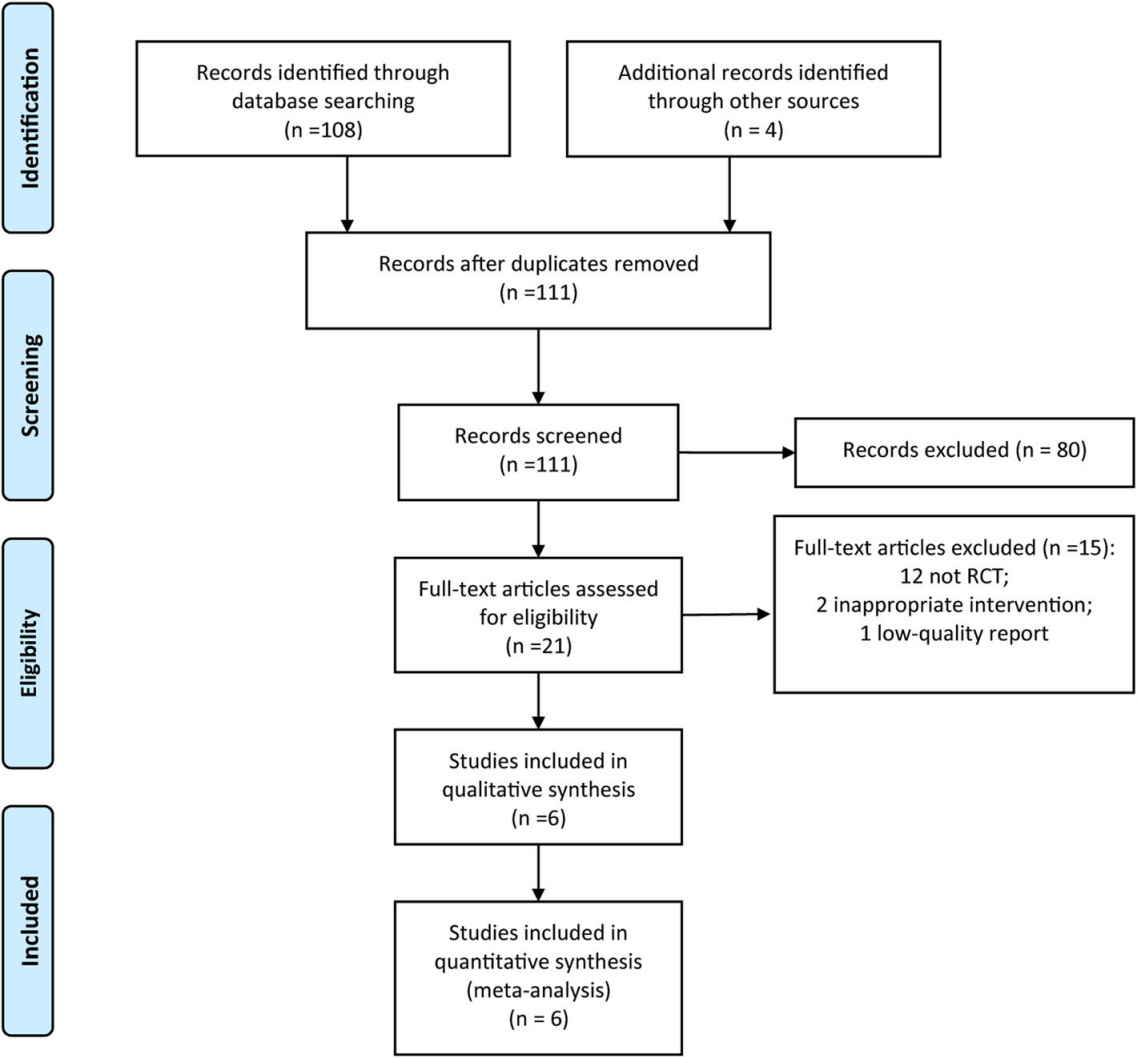
The flow chart of study selection

Figure 1 shows a flow diagram of study selection. The electronic searches identified 111 literatures, and 80 reports were clearly not relevant after the first screening. The full text of 21 studies was retrieved for further in-depth consideration, and 15 studies were excluded for several reasons: 12 studies were not RCT, 2 studies were with inappropriate intervention, and 1 study was a low-quality report. Finally, 6 RCTs [18–23] were included in this meta-analysis. References cited in published original and review papers were examined, and no further studies were found.

### Characteristics of included RCTs

The characteristics of RCTs included in this present meta-analysis are presented in Table 1. Of these included studies, one was performed in India [23], and the rest of the five studies in China [18–22]. The combined sample size across the 6 included studies was 277 participants, with 142 ALI patients with omega-3 fatty treatment and 135 patients without omega-3 fatty acid treatment. The patient samples in the included studies were different with a range of 34 to 61. For study results, one study did not support the use of omega-3 fatty in ALI patients, whereas the other studies all favored the use of omega-3 fatty in the treatment of ALI.

**Table 1:** The characteristics of the included RCTs

Studies	Countries	Group	Participants	Age (years)	Length of diet	Conclusions
Dai 2009	China	Omega-3 fatty acids	17	39.5 ± 5.8	1 week	Omega-3 fatty acids are beneficial to the recovery of respiratory function in patients with ARDS
		Control	17			
Gupta 2010	India	Omega-3 fatty acids	31	51.16 ± 15.58	1 week	In ventilated patients with ARDS, intravenous omega-3 fatty acids alone do not improve ventilation, length of ICU stay, or survival
		Control	30	46.63 ± 16.44		
Shen 2010	China	Omega-3 fatty acids	20	66.1 ± 15	1 week	Omega-3 polyunsaturated fatty acid improves respiratory function and prognosis in patients with ALI.
		Control	19	61.9 ± 18.9		
Shen 2011	China	Omega-3 fatty acids	22	64.0 ± 9.0	1 week	The use of omega-3 polyunsaturated fatty acids helps to improve inflammatory factor levels in ALI patients.
		Control	21	68.0 ± 10.0		
Wang 2011	China	Omega-3 fatty acids	26	75.2 ± 8.9	1 week	Omega-3 fatty acids are beneficial to the recovery of respiratory function in ALI patients, and it shortens the time of mechanical ventilation and length of ICU stay.
		Control	22			
Zhao 2010	China	Omega-3 fatty acids	26	40.2 ± 6.1	1 week	The omega-3 polyunsaturated fatty acids enriched enteral nutrition solution can facilitate the recovery of respiratory function.
		Control	26			

*ARDS* acute respiratory distress syndrome, *ALI* acute lung injury

### Methodological quality of the included studies

**Fig. 2:**
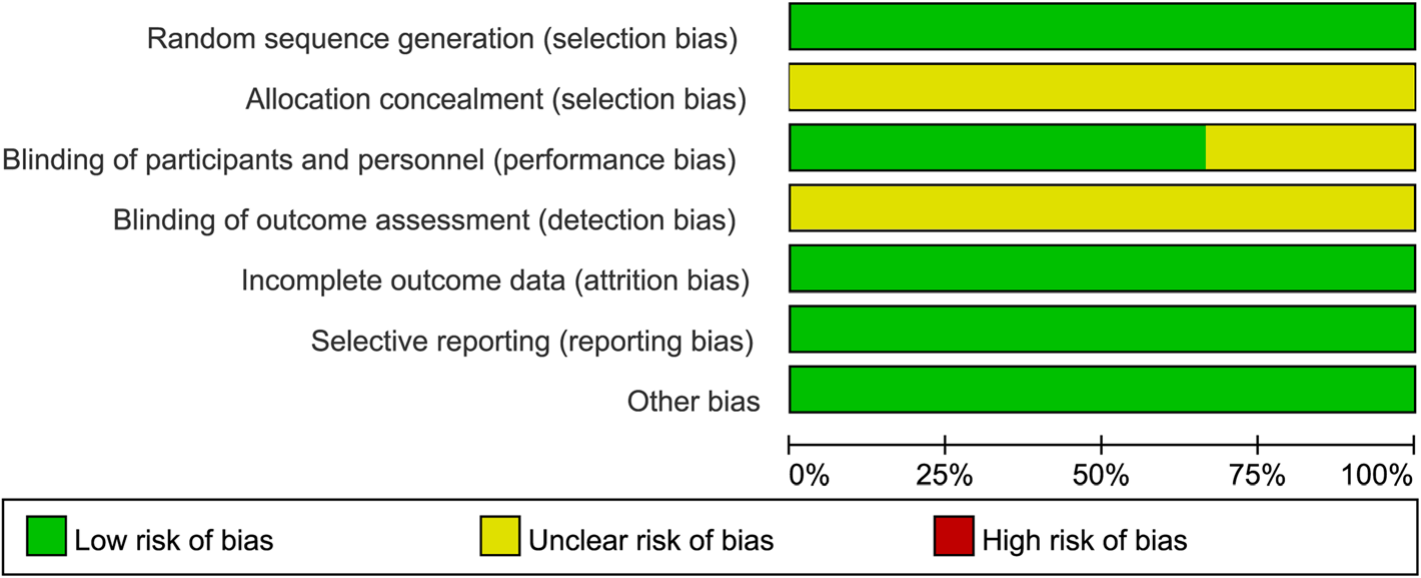
Risk of bias graph

**Fig. 3:**
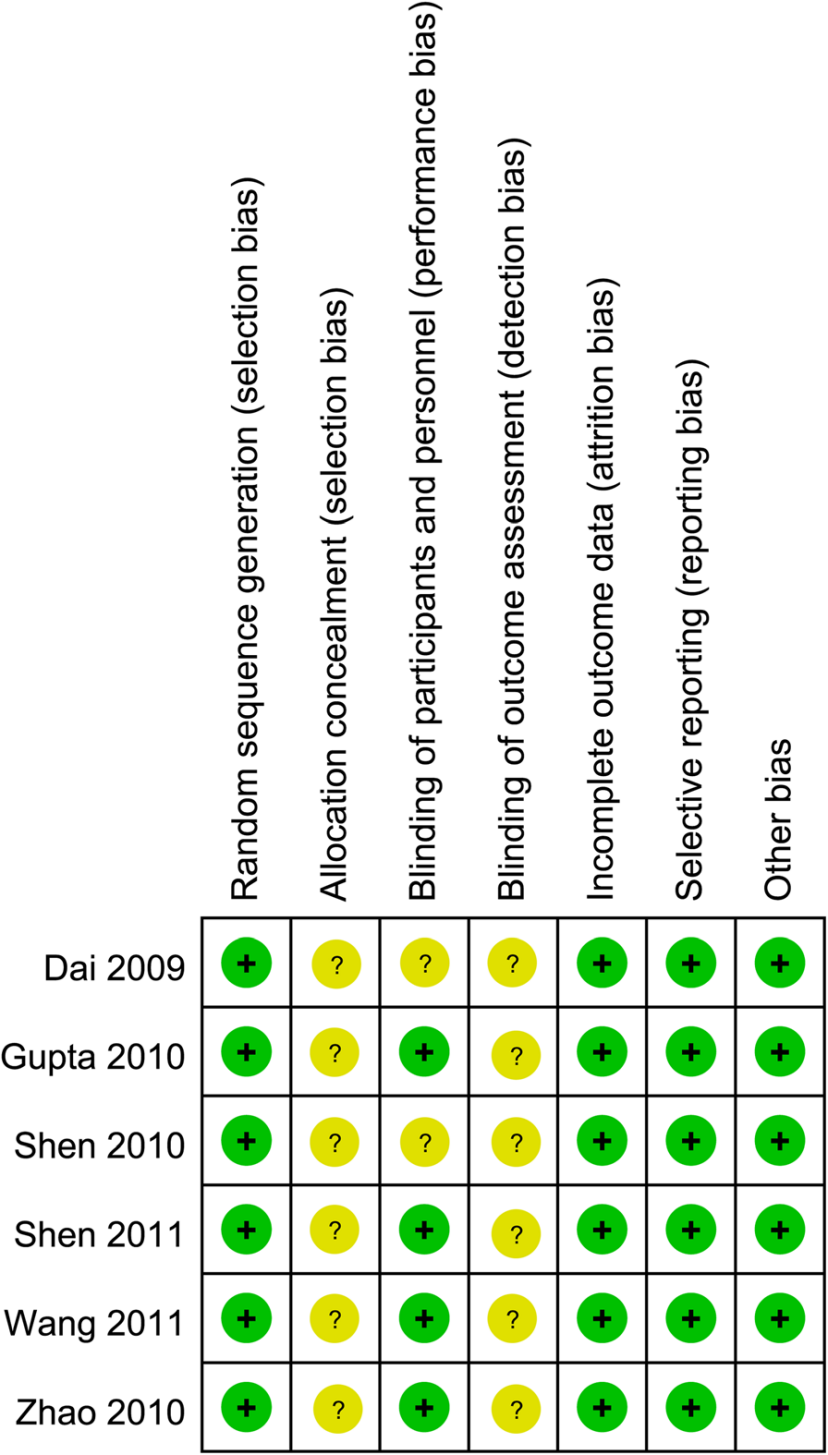
Risk of bias summary

Figures 2 and 3 show the quality assessment of the studies in this present meta-analysis. All 6 included studies were randomized, but only two RCTs [21, 23] specified the method of randomization. All the RCTs did not report whether any allocation concealment process was used. Four RCTs [20–23] reported the design on blinding patients, personnel, and outcome assessors. No other significant biases among the included RCTs were found. Intention to treat (ITT) was performed for all patients.

### Outcomes

#### The changes of PaO_2_

**Fig. 4:**
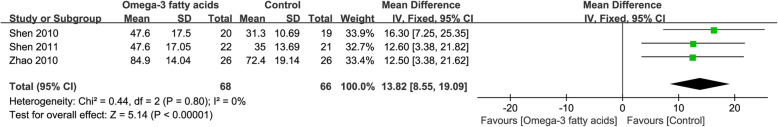
Forest plot for the changes of PaO_2_

Three RCTs [19, 20, 22] reported the changes of PaO_2_ for ALI patients with or without omega-3 fatty acid treatment; the pooled data from the three RCTs revealed that the omega-3 fatty acid treatments significantly improve the PaO_2_ in ALI patients (MD = 13.82, 95% CI 8.55–19.09, *P* < 0.001, *I*^2^ = 0%; Fig. 4).

#### The changes of PaO_2_/FiO_2_

**Fig. 5:**
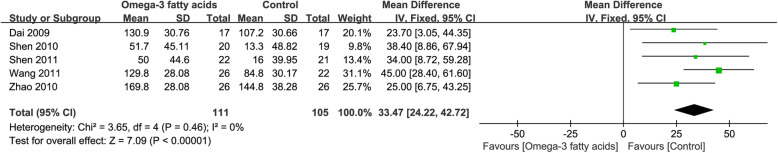
Forest plot for the changes of PaO_2_/FiO_2_

Five RCTs [18–22] reported the changes of PaO_2_/FiO_2_ for ALI patients with or without omega-3 fatty treatment; the pooled data from the five RCTs revealed that the omega-3 fatty acid treatments significantly improve the PaO_2_/FiO_2_ in ALI patients (MD = 33.47, 95% CI 24.22–42.72, *P* < 0.001, *I*^2^ = 0%; Fig. 5).

#### The changes of total protein

**Fig. 6:**
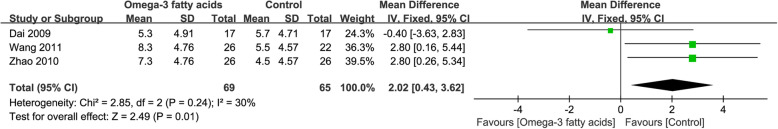
Forest plot for the changes of TP

Three RCTs [18, 21, 22] reported the changes of TP for ALI patients with or without omega-3 fatty treatment; the pooled data from the three RCTs revealed that the omega-3 fatty acid treatments significantly increase the TP in ALI patients (MD = 2.02, 95% CI 0.43–3.62, *P* = 0.01, *I*^2^ = 30%; Fig. 6).

#### The duration of mechanical ventilation

**Fig. 7:**
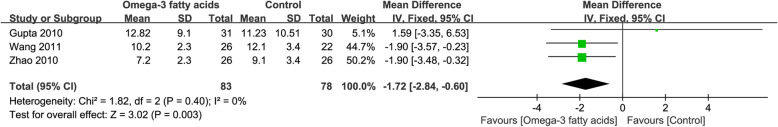
Forest plot for the duration of MV

Three RCTs [21–23] reported the duration of MV for ALI patients with or without omega-3 fatty treatment; the pooled data from the three RCTs revealed that the omega-3 fatty acid treatments significantly reduced the duration of MV in ALI patients (MD = − 1.72, 95% CI − 2.84 to − 0.60, *P* = 0.003, *I*^2^ = 0%; Fig. 7).

#### The length of ICU stay

**Fig. 8:**
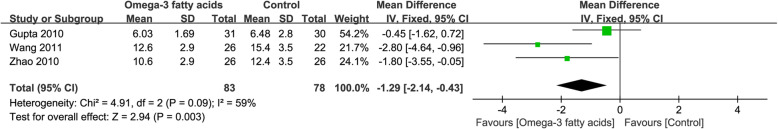
Forest plot for the length of ICU stay

Three RCTs [21–23] reported the length of ICU stay for ALI patients with or without omega-3 fatty acid treatment; the pooled data from the three RCTs revealed that the omega-3 fatty acid treatments significantly reduced the length of ICU stay in ALI patients (MD = − 1.29, 95% CI − 2.14 to − 0.43, *P* = 0.003, *I*^2^ = 59%; Fig. 8).

### Subgroup and sensitivity analyses

No subgroup analyses were performed in our study because the heterogeneity among the included RCTs in the synthesized results remained small. We attempted to evaluate publication bias by using a funnel plot if 10 or more RCTs were included [24]. However, the number of included RCTs was only six; we could not evaluate publication bias by using a funnel plot. Sensitivity analyses, which investigate the influence of one study on the overall risk estimate by removing one study in each turn, suggested that the overall risk estimates were not substantially changed by any single study.

## Discussion

Patients with ALI are in a state of high metabolism, and it is necessary to provide nutritional support [25]. Besides, the nutritional support of critically ill patients should fully consider the tolerance of the damaged organ; critically ill patients should start EN as soon as the conditions permit [25]. It has been reported that early EN support improves immune function and protein levels in ARDS patients, which may reduce mortality and shorten the duration of MV and ICU stay [26]. With six RCTs included, the results of this present meta-analysis have indicated that omega-3 fatty acids significantly improve the PaO_2_, PaO_2_/FiO_2_ increase the TP level, and reduce the length of MV and ICU stay for ALI patients. Therefore, the use of omega-3 fatty acids is a promising and effective treatment strategy for ALI.

The potential mechanism of omega-3 fatty acids for ALI treatment should be considered. A number of studies [27–29] have found that omega-3 fatty acids can regulate the synthesis of lipid mediators, release cytokines, and activate white blood cells and endothelial cells, thereby regulating the body’s excessive inflammatory response to reduce the lung inflammation. Moreover, it is possible that omega-3 fatty acids increase the patient’s PaO_2_ and PaO_2_/FiO_2_ to improve the patient’s respiratory function. Furthermore, omega-3 fatty acids can reduce the concentration of IL-8, leukotriene, and neutrophils in the bronchial lavage fluid of patients, and reduce alveolar permeability, thereby improving patients’ gas exchange [30, 31]. Meanwhile, it not only provides energy as a nutrient-supporting substance and maintains nitrogen balance, but also reduces insulin resistance and hyperlipidemia [32]. However, the potential risks of omega-3 fatty acid use should also be concerned. Two included RCTs [21, 22] reported the adverse events after the use of omega-3 fatty, mainly diarrhea, bloating, and gastric contents reflux. It can be related to that after long-term or unreasonable use of antibiotics, the intestinal flora is dysfunctional, which can easily lead to diarrhe a[33]. And it is possible that the nutrient solution temperature is too low [34]. The occurrence of diarrhea is negatively associated with the serum protein levels [35]. Attention should be paid to the speed of infusion; the temperature of the nutrient solution should also be appropriate.

The pathological feature of ALI is pulmonary edema caused by increased pulmonary capillary permeability; the pathological basis of which is neutrophil-mediated local inflammatory response in the lung [36]. Pathophysiological changes were mainly due to increased Qs/Qt and imbalance of ventilation/blood flow ratio [37]. The application of omega-3 fatty acids not only can quickly reduce the inflammatory reaction of lung tissue, but also can transform LTB4, which aggravates inflammatory reaction, into LTB5 series, which is less active, thereby significantly reducing pulmonary edema, improving the pulmonary vascular permeability [38–40]. Besides, as specific immunonutrients, omega-3 fatty can regulate the activity of lipid mediators, cytokines, and endothelial cell, thereby regulating the excessive inflammatory response of the body under infection and trauma [41]. Given all this together, the use of omega-3 fatty may be a useful adjuvant therapy for ALI patients.

The limitations existed in this study must be considered. Firstly, the number of RCTs included in this study is too small, and five out of six RCTs were from China, and the representativeness is poor. Secondly, the potential risk of bias in the allocation concealment process, blinding of researchers, and blinding of outcome assessments must be considered since the information on those issues remains unclear; omega-3 fatty treatment was associated with the reduced days of ICU for ALI patients, but the result should be taken cautiously. Further studies with strict design are needed. Thirdly, no relevant economic indicators were reported among included RCTs; thus, we could not perform analysis on this issue. Future clinical trials should focus on collecting and reporting relevant economic data to evaluate the cost of omega-3 fatty acid treatment. And the selected indicators should reflect the purpose of the study as much as possible with longer follow-up period.

In conclusion, as a kind of nutritional preparation, omega-3 acids can improve the respiratory function and respiratory status of ALI patients by increasing PaO_2_ and PaO_2_/FiO_2_. Meanwhile, it can increase the TP level, and reduce the length of MV and ICU stay of ALI patients. Therefore, omega-3 fatty may be a potential effective and safe strategy for the ALI treatment. Future larger-scale RCTs are needed to further evaluate the timing and dosage of omega-3 fatty application in ALI treatment.

## Data Availability

All data generated or analyzed during this study are included in this published article.

## Abbreviations

RCTs: Randomized controlled trials
ALI: Acute lung injury
OR: Odds ratios
MD: Mean differences
CI: Confidence intervals
ARDS: Acute respiratory distress syndrome
CNKI: China National Knowledge Infrastructure
CBM: China Biomedical Literature Database
ITT: Intention to treat
TP: Total protein
MV: Mechanical ventilation
PUFA: Polyunsaturated fatty acids
